# Vitamin D Status in Rheumatology Patients with Inflammatory Compared with Non-Inflammatory Diagnoses: Inflammatory and Autoimmune Markers Are Not Associated with Vitamin D Levels

**DOI:** 10.3390/nu18020326

**Published:** 2026-01-20

**Authors:** Arne Schäfer, Magdolna Szilvia Kovacs, Axel Nigg, Martin Feuchtenberger

**Affiliations:** 1 Medizinische Klinik und Poliklinik II, University Hospital Würzburg, 97080 Wuerzburg, Germany; 2 Diabetes Zentrum Mergentheim, 97980 Bad Mergentheim, Germany; 3 Rheumatologie, Medizinisches Versorgungszentrum (MVZ) MED BAYERN OST, 84489 Burghausen, Germany

**Keywords:** vitamin D, inflammatory rheumatic disease, epidemiology, rheumatology, electronic health records, C-reactive protein, rheumatoid factor

## Abstract

Background/Objectives: Vitamin D levels tend to be lower in patients with inflammatory rheumatic diseases (IRDs), including rheumatoid arthritis (RA), but there are minimal data on vitamin D levels in rheumatology patients with inflammatory vs. non-inflammatory diagnoses. Methods: In this retrospective, observational study, we used electronic health record data from patients presenting for their first visit at a large rheumatology clinic to assess vitamin D levels and deficiency based on diagnosis, and to evaluate the association between vitamin D and inflammatory markers (including C-reactive protein [CRP]) or autoimmune markers (including rheumatoid factor [RF], anti-citrullinated peptide antibody, and anti-nuclear antibodies). Logistic regression analysis with 13 clinical variables was used to evaluate the association between vitamin D levels and IRD diagnosis, and linear regression was used to evaluate the association between vitamin D levels and CRP or RF. Results: The patient cohort included 4979 patients; 1385 (27.8%) had an IRD. Vitamin D levels were significantly lower in the IRD vs. non-inflammatory subgroup (mean [SD] of 26.6 [13.3] vs. 27.7 [14.3]; *p* = 0.009), but the difference was not clinically relevant given the small effect size. Vitamin D deficiency rates (<20 ng/mL) were not significantly different between the subgroups, and vitamin D was not associated with an IRD diagnosis in logistic regression analysis. In linear regression analysis, vitamin D was not associated with CRP or RF in the full patient cohort or in the subgroup with RA (n = 539). Conclusions: We conclude that vitamin D levels do not differ substantially based on IRD versus non-inflammatory diagnosis, CRP levels, or RF levels in this clinical cohort.

## 1. Introduction

Vitamin D plays a key role in musculoskeletal health and immune function, and both of these systems are impacted by inflammatory rheumatic diseases (IRDs) such as rheumatoid arthritis (RA) and spondyloarthropathies (SpA) [1,2]. By definition, IRDs affect various parts of the musculoskeletal system, particularly joints and muscles, although other organs may also be involved [1]. IRDs are also associated with a dysregulated immune response, and in many cases, pathogenesis appears to be driven by the immune system [3,4]. The most common IRD, RA, is characterized by immune-mediated joint inflammation, and the majority of patients have autoantibodies, such as rheumatoid factor (RF), anti-citrullinated peptide antibody (ACPA), and, less frequently, anti-nuclear antibodies (ANA) [5].

A number of reports have documented associations between vitamin D levels and IRDs, although potential mechanisms for this have not yet been delineated. Many studies have found higher rates of vitamin D deficiency or lower vitamin D levels in patients with IRDs compared with control groups [6,7,8,9,10,11,12], whereas others have reported an association between lower vitamin D levels and higher disease activity [6,7,8,13,14]. The effectiveness of vitamin D supplementation in reducing IRD symptoms and disease activity is unclear and has been described as “still a challenge” [15], with one recent systematic review and meta-analysis finding positive effects [16] and another finding no evidence of benefit [17].

To the best of our knowledge, there is only one published study comparing vitamin D levels in patients with IRDs to rheumatologic patients with non-inflammatory musculoskeletal diagnoses. This study, which included 81 patients with IRDs and 26 patients with non-inflammatory diagnoses, did not identify a significant difference between these two groups [18]. However, the small size of this study suggests that additional analyses might be of value.

We previously used electronic health record (EHR) data from a large patient cohort seen at a rheumatology clinic in southern Germany to evaluate vitamin D levels and patient characteristics [19]. The study reported here extends these initial observations and evaluates vitamin D levels and patient characteristics based on inflammatory vs. non-inflammatory diagnoses. The goal of this study was to assess whether vitamin D levels and status varied based on diagnosis and whether vitamin D levels were associated with inflammatory or autoimmune markers in patients presenting to a rheumatology clinic. We hypothesized that if systemic inflammation is associated with vitamin D deficiency, then vitamin D levels should be significantly lower in patients with systemic inflammation due to IRDs compared with those with non-inflammatory musculoskeletal diagnoses, independent of other factors. We intentionally chose the time point of initial presentation (first visit) for these analyses, as this represents the “diagnostic window” where patients are typically symptomatic and treatment-naïve.

## 2. Materials and Methods

### 2.1. Study Design

We conducted a retrospective cross-sectional cohort study based on EHR data from patients presenting for the first time to a single large secondary care center specializing in rheumatology in Burghausen, Germany. Patients were seen at the clinic between 1 January 2021 and 31 December 2024. The first patient visit was chosen for this analysis because patients at our clinic are typically treatment-naïve at this point, thereby eliminating a potential confounding factor. Blood tests for vitamin D levels (as described below) were performed in all patients as part of routine clinical care. At this center, vitamin D measurement is performed only at initial presentation unless clinical signs indicate the need for additional investigation. Any subsequent vitamin D test results were not included in this study. Variables of interest included patient characteristics, vitamin D assay results, laboratory markers, patient-reported outcomes (PROs), and final diagnosis. This study was approved by the Institutional Review Board (IRB) of Würzburg University (#207/21-me). Due to the retrospective design and use of de-identified patient data, the study received a waiver for individual patient consent from the IRB. All research activities were conducted in accordance with the ethical principles outlined in the Declaration of Helsinki.

Generative artificial intelligence was not used to conduct this study or in the preparation of the manuscript.

### 2.2. Assays for Vitamin D and Inflammatory Markers

The Elecsys^®^ Vitamin D total III assay (Roche Diagnostics GmbH, Mannheim, Germany), an electrochemiluminescence binding assay that measures 25(OH)D (both 25-hydroxyvitamin D2 and 25-hydroxyvitamin D3) in human plasma and serum [20], was used to measure vitamin D levels. Assays were performed at the clinic site. Varying definitions are applied to vitamin D deficiency/sufficiency categorizations [21], but for the purposes of this study deficient vitamin D levels were defined as <20 ng/mL (<50 nmol/L), sufficient as 20–30 ng/mL (50–75 nmol/L), optimal as >30–70 ng/mL (>75–175 nmol/L), and elevated as >70 ng/mL (>175 ng/mL) [19].

Commercial assays were used to evaluate serum C-reactive protein (CRP), erythrocyte sedimentation rate (ESR), ANA/extractable nuclear antigen (ENA) positivity (Euroimmun, Lübeck, Germany) and RF and ACPA IgG titers (both Roche Diagnostics, Indianapolis, IN, USA). ANA/ENA tests utilized enzyme-linked immunoassays (ELISAs) for IgG antibodies to dsDNA, histones, ribosomal P proteins, ribonucleoproteins/Smith antigen, SS-A, SS-B, Scl-70, Jo-1, and centromeres with cut-offs for positive/negative results as recommended by the manufacturer. Assays were performed at a single central laboratory.

### 2.3. Patient-Reported Outcomes

PROS evaluated in this study included the Pain-visual analog scale (Pain-VAS; scale of 0 to 100), patient assessment of global disease activity (PtGA)-VAS (scale of 0 to 100), Patient Health Questionnaire-2 (PHQ-2; score of 0 to 6), and the fibromyalgia Symptom Severity Scale score (SSS; score of 0 to 12).

### 2.4. Statistical Analysis

This study was a retrospective evaluation that used an existing dataset to explore general trends and associations in patients presenting for the first time to a rheumatology center. Analyses were based on data obtained at a single visit for each patient; longitudinal data were not evaluated. The focus of this study was on generating data to allow future in-depth evaluations, rather than on evaluating specific hypotheses with pre-defined error rates. All eligible patients who met entry criteria were included in this study. Due to the explorative nature of the study, sample size calculations were not performed.

Patient characteristics and laboratory values were summarized through descriptive statistics, including mean, standard deviation (SD), and percentages. Between-group differences in age and vitamin D levels were evaluated by t-tests, and Pearson chi-square tests were used to assess between-group differences in categorical variables (e.g., sex and vitamin D status). Bivariate associations among different variables, including vitamin D levels, inflammatory markers, and PROs, were evaluated by Pearson correlation analysis with corresponding correlation coefficients (*r*).

Logistic regression analysis was used to evaluate the association between vitamin D levels and inflammatory (vs. non-inflammatory) diagnosis in the full patient cohort. The following variables were included: age, male sex, vitamin D levels (ng/mL), Pain-VAS score, PtGA score, PHQ-2 score, SSS score, RF titer (units/mL), ACPA titer (units/mL), ANA/ENA positivity, CRP (mg/dL), ESR (mm/h), and leukocyte count (×10^3^ per µL). These variables were empirically chosen due to clinical relevance in IRDs.

The associations between vitamin D levels and key inflammatory/autoimmune markers (CRP and RF) were evaluated by linear regression using the same variables as in the logistic regression analysis. These analyses were considered secondary explorations as they were based on laboratory markers rather than on clinical diagnosis. While markers such as CRP and RF typically exhibit right-skewed distributions, linear regression models were conducted without logarithmic transformation. Given the large sample size, the Central Limit Theorem [22] ensures the robustness of the regression estimates against violations of normality, and the use of raw units preserves clinical interpretability. Linear regression analyses were conducted in both the overall cohort and the subset of patients with RA. Pearson correlation analyses were used to evaluate model fit and statistical significance.

Analyses of statistical significance were two-sided unless otherwise specified, and *p* < 0.05 was considered statistically significant. Statistical testing and data analyses were conducted using SPSS for Windows, version 29.0 (IBM Corp., Armonk, NY, USA).

## 3. Results

### 3.1. Demographic Characteristics and Vitamin D Status in Inflammatory vs. Non-Inflammatory Rheumatologic Diagnoses

As reported previously, 4979 patients were included in these analyses (64.9% female, mean [SD] age 53.6 [15.2] years) [19]. Most patients (3594/4979 [72.2%] had a non-inflammatory diagnosis, usually osteoarthritis, and the remainder (1385/4979 [27.8%] had an IRD, most commonly rheumatoid arthritis (RA; n = 539) (Table 1). The subgroup of patients with IRDs was significantly older than the subgroup with non-inflammatory diagnoses (mean [SD] of 58.6 [15.7] vs. 51.7 [14.6] years; *t*-test *p* < 0.001) and had a significantly higher percentage of males (52.1% vs. 28.6%; Pearson chi-square *p* < 0.001). The IRD subgroup also had significantly lower mean vitamin D levels compared with the non-inflammatory subgroup (mean [SD] of 26.6 [13.3] vs. 27.7 [14.3] ng/mL; *t*-test *p* = 0.009), but SD values were large and the effect size was small (Cohen’s *d* = 0.083).

**Figure 1:**
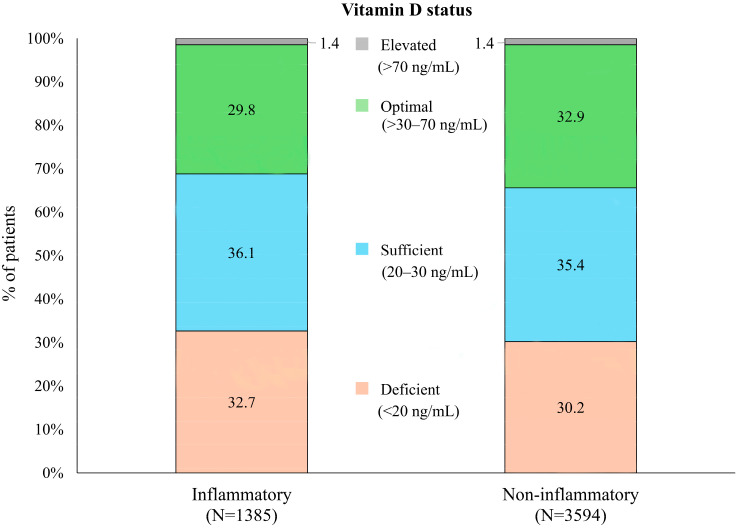
Distribution of vitamin D status in patients with inflammatory vs. non-inflammatory rheumatologic diagnoses.

In analyses of vitamin D status, there was a higher percentage of individuals with vitamin D deficiency (<20 ng/mL) in the inflammatory (453/1385; 32.7%) vs. non-inflammatory (1087/3594; 30.2%) subgroups, while optimal levels (>30 to 70 ng/mL) were lower in patients with inflammatory diagnoses (Figure 1). However, differences were minimal, and the association between diagnosis and categorized vitamin D status failed to achieve statistical significance (Pearson chi-square *p* = 0.154).

Among patients with IRDs, those with various types of inflammatory arthritis had the highest proportions of patients with vitamin D deficiency. Specifically, the rate of vitamin D deficiency in the subgroup with other forms of inflammatory arthritis (non-RA/non-SpA) was 39.8% (78/196), axial SpA was 35.7% (35/98), and RA was 34.0% (183/539) (Supplementary Table S1). Patients with connective tissue disease (25.0% [22/88]) and “other” IRDs, including polymyalgia rheumatica (27.6% [81/293]), had the lowest rates of vitamin D deficiency. The difference in vitamin D status among varying IRD diagnoses was not statistically significant (Pearson chi-square *p* = 0.187).

### 3.2. Bivariate Associations Between Vitamin D and Key Clinical Variables

**Figure 2:**
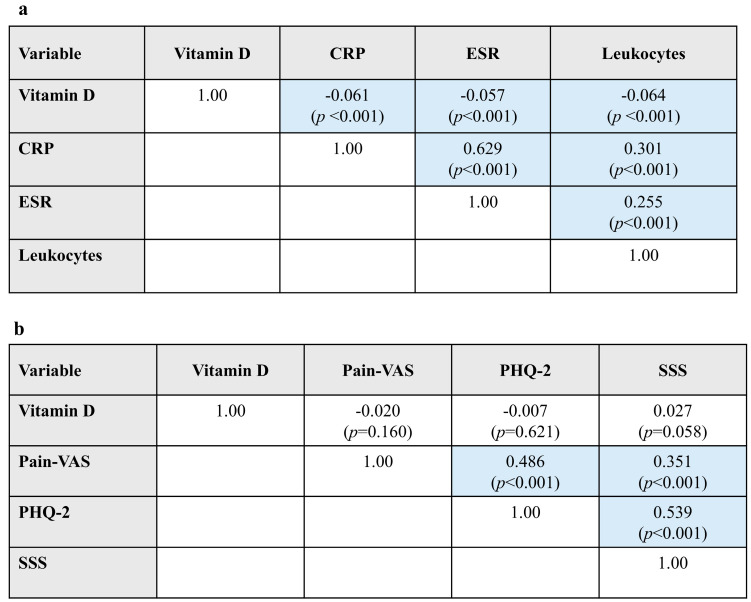
Bivariate associations between vitamin D and (**a**) laboratory variables and (**b**) PROs in Pearson correlation analyses. Data are presented as Pearson *r* correlation coefficients (*p*-value). Blue shading indicates statistically significant results. CRP, C-reactive protein; ESR, erythrocyte sedimentation rate; PHQ, Patient Health Questionnaire; PRO, patient-reported outcome; SSS, Symptom Severity Scale score for fibromyalgia; VAS, visual analog scale.

In the full patient cohort (N = 4979), Pearson correlation analyses found that higher vitamin D levels were significantly associated with lower levels of inflammatory markers (CRP, ESR, and leukocytes), but effect sizes (correlation coefficients) were small (*r* < |0.1|) (Figure 2a). As expected, CRP and ESR showed a strong positive correlation with each other and were moderately correlated with leukocyte counts, another marker of inflammation.

Vitamin D levels showed no significant associations with PROs (Pain-VAS, PHQ-2, or SSS). Moderate positive correlations were observed between pain and PHQ-2, pain and SSS, and PHQ-2 and SSS (Figure 2b).

### 3.3. Logistic Regression Analysis of Variables Influencing Inflammatory Diagnoses

To identify clinical variables that may be associated with IRDs, we performed a logistic regression analysis of the full cohort (see Section 2.4 for included variables). Because the data for this study were obtained from a single center with a uniform protocol for data collection at the first visit, there were no missing values for evaluated variables. The model explained 30.5% of the variability in IRD diagnoses (Cox and Snell *R*^2^ = 0.305). In the logistic regression analysis, vitamin D was not significantly associated with IRD (Wald *p* = 0.100). All other variables were significantly associated with IRD, but the odds ratios generally indicated minimal effects except for ANA/ENA (exp B = 8.672), male sex (3.320), CRP (1.493), and leukocyte counts (1.221) (Table 2). Although some of the parameters in the model were intercorrelated, such as CRP and ESR, the small standard errors indicate that the model is mathematically stable.

In correlation matrix analysis, vitamin D levels showed only weak correlations with other variables, including auto-antibodies (RF, ACPA, and ANA/ENA) and markers of inflammation (CRP, ESR, and leukocytes). The variable most strongly correlated with vitamin D was male sex (*r* = 0.113) (Supplementary Figure S1). The strongest correlations between other variables were Pain-VAS and PtGA-VAS (*r* = −0.583), CRP and ESR (*r* = −0.493), and PHQ-2 and SSS (*r* = −0.477).

### 3.4. Linear Regression Analysis of Associations Between Vitamin D Levels and Clinical Variables

We conducted a linear regression analysis of associations between vitamin D and laboratory markers using the same variables as in the logistic regression analysis described previously. Separate analyses were conducted with CRP as the dependent variable and with RF as the dependent variable. We conducted these secondary analyses to supplement the logistic regression analyses based on clinical diagnosis and to further explore the possible association between vitamin D levels and inflammatory or autoimmune markers.

In the full cohort (N = 4975), the included variables explained 43.9% of the observed variance in CRP values (model fit *R*^2^ = 0.439). Vitamin D had a β coefficient of <−0.001 (*p* = 0.670), indicating that it was not significantly associated with CRP levels. Significant associations with CRP were male sex, Pain-VAS, PtGA-VAS, ANA/ENA, ESR, and leukocyte counts (Table 3).

For RF in the full cohort, the model explained only 16.2% of the variance in values (*R*^2^ = 0.162). Vitamin D was not significantly associated with RF values (β = −0.065; *p* = 0.187). Significant associations for RF were age, ACPA, ESR, and leukocyte counts (Table 3).

Because it was possible that the large number of patients with non-inflammatory diagnoses obscured associations between vitamin D and laboratory markers, we repeated the linear regression analyses in the subgroup of patients with RA (n = 539). This resulted in an increase in the model fit (*R*^2^ = 0.515) for CRP, but, as in the full cohort analysis, vitamin D was not significantly associated with CRP (β = −0.005; *p* = 0.432). Variables associated with CRP levels in RA patients were male sex, PtGA-VAS, ESR, and leukocyte counts. In analyses of RF, confining the dataset to RA patients did not improve the model fit (*R*^2^ = 0.118). Vitamin D was not significantly associated with RF (β = −0.352; *p* = 0.427). The only variables associated with RF levels in the RA subgroup were ACPA and leukocyte counts (Table 3).

## 4. Discussion

To the best of our knowledge, this is the largest study to assess vitamin D levels in inflammatory vs. non-inflammatory rheumatology patients and to evaluate possible associations with multiple inflammatory and autoimmune markers. In this large cohort of patients presenting to a rheumatology clinic, patients with an IRD had significantly lower mean vitamin D levels than those with a non-inflammatory diagnosis, but the difference was small, and there was no significant difference in the rate of vitamin D deficiency between patients with and without an IRD. Logistic regression analysis with 13 variables also failed to identify a significant association between vitamin D levels and an IRD diagnosis. In supplementary linear regression models, vitamin D levels were not associated with CRP levels, a key marker of inflammation, or RF titers, a marker of autoimmunity, in either the full patient cohort or the subgroup of patients with RA. These findings were consistent with correlation matrix analysis for variables potentially associated with IRDs, which also failed to identify correlations between vitamin D levels and markers of inflammation (CRP, ESR, or leukocytes) or between vitamin D and auto-antibodies (RF, ACPA, or ANA/ENA). On the basis of these findings, we conclude that lower vitamin D levels are not associated with IRD diagnosis or CRP or RF levels to a clinically relevant extent in patients presenting to a rheumatology clinic.

The association between low vitamin D levels and IRDs is a consistent observation across most studies [6,7,8,9,10,11,12], including the study reported here, but there are some exceptions [23,24,25]. Although it is tempting to try to connect the lower vitamin D levels observed in patients with IRDs to inflammatory disease processes, vitamin D levels are affected by a myriad of factors, including sun exposure, skin pigmentation, body mass index (BMI), metabolic differences, and inter-individual variations in gene expression in response to vitamin D [26,27,28]. In our study, the difference in mean vitamin D levels between the IRD and non-inflammatory diagnosis group was minor and did not retain significance in a logistic regression analysis. In our previous study of the same patient cohort, younger age and male sex were associated with lower vitamin D levels [19]. It is thus possible that the significantly higher percentage of male patients in the IRD subgroup contributed to the lower vitamin D levels observed in the bivariate analysis, but not the logistic regression analysis, which adjusted for sex. In a cohort of this size, which can result in detection of statistically significant associations despite negligible effect sizes, the failure to identify an association between vitamin D and IRD diagnosis in the logistic regression analysis strongly supports the absence of a clinically relevant association between these two variables.

The large size of the study cohort allowed us to systematically analyze the association between vitamin D and individual inflammatory and autoimmune markers, including CRP and RF. It is important to note that both inflammation and autoimmunity involve complex pathways, and therefore, none of these laboratory markers should be considered in an isolated manner. Although there was a significant bivariate association between CRP and vitamin D, the effect size was small. In linear regression analyses, vitamin D levels did not contribute significantly to the modeling of CRP or RF levels, either in the full patient cohort or in the subgroup of patients with RA. Haque et al. [29] came to a similar conclusion concerning the association between vitamin D and CRP in a study of patients with RA and cardiovascular risk factors. In their study, the association between these variables observed in unadjusted models was no longer significant in adjusted models that included sex, ethnicity, season, and BMI. A different conclusion was reached in a large study conducted in men with RA, which found a significant association between vitamin D deficiency (<20 ng/mL) and higher CRP concentrations [13]. An evaluation of data from the randomized VITAL trial, in which vitamin D3 supplementation was associated with a reduced risk of autoimmune disease [30], suggested that the effect of vitamin D on CRP levels may be transient [31]. In this study, vitamin D supplementation was associated with a statistically significant reduction in CRP levels at 2 years, but not at 4 years [31]. There is currently no evidence that vitamin D is associated with RF; consistent with our findings, one study failed to detect a difference in vitamin D status between RF-positive and RF-negative patients [13]. However, associations between vitamin D and other auto-antibodies, specifically ACPA [13] and ANA [32], have been reported in other studies. In our study, correlation analyses of variables included in the logistic regression analysis did not detect significant associations between vitamin D and auto-antibodies.

Evaluations of potential “drivers” of IRD are complicated by the fact that vitamin D, CRP, and RF are all affected by multiple factors [26,27,28,33,34], and so more complex models may be needed to further explore possible associations between vitamin D and IRDs. Some of the other mechanisms that have been proposed to explain the presence of lower vitamin D levels in patients with IRDs include effects on myeloid-derived cytokines such as TNF and IL-6, modulation of synovial-derived molecules such as matrix metalloprotease-1, alterations in T-cell differentiation, regulation of antigen-presenting cells, and effects on antibody production by B lymphocytes [1,5].

Although vitamin D was not associated with IRD diagnosis in this study, most of the other laboratory and PRO variables evaluated did show a significant association with IRD status, although effect sizes were generally small. The exceptions were ANA/ENA positivity (β coefficient = 8.672) and male sex (β coefficient = 3.320). Correlation analyses of variables included in the logistic regression model for IRDs detected known associations, such as between Pain-VAS and PtGA-VAS and between CRP and ESR, thus supporting the validity of these analyses, but did not identify associations between vitamin D levels and any of the other variables, including auto-antibodies, inflammatory markers, or PROs.

Our study has the limitations inherent to retrospective, observational studies, including a non-randomized, single-center patient cohort. The study reported here did not include “healthy” controls, as all patients were being seen at a rheumatology clinic. Although inflammatory processes do not drive the pathogenesis of non-inflammatory diagnoses, patients with non-inflammatory diagnoses may still be experiencing inflammation, either related to or distinct from their rheumatologic condition. A previous smaller study that compared vitamin D levels in patients with IRDs to those with non-inflammatory musculoskeletal diagnoses and healthy controls found that patients with IRDs had significantly lower vitamin D levels than healthy controls, but there was no significant difference between vitamin D levels in the inflammatory vs. non-inflammatory patient group [18]. Information on vitamin D supplementation or dietary sources of vitamin D was not reported in the EHR; both are known to influence vitamin D levels [26,35]. Other variables that can influence vitamin D levels and are not available in EHR data include sun exposure, diet, physical activity, and socioeconomic status. We consider it unlikely that these variables would vary systematically between patients with IRDs vs. non-inflammatory diagnoses, but acknowledge the potential impact of residual confounding from these variables. BMI and seasonality were analyzed previously [19] and did not differ significantly between groups. Moreover, with respect to seasonality, given the continuous recruitment over a 4-year period (2021–2024), seasonal variations are expected to be distributed equally between the inflammatory and non-inflammatory diagnoses, thereby mitigating the risk of systematic seasonal bias. The logistic regression model used in our study included several variables, including RF, ACPA, and CRP, that are closely linked to the diagnosis of IRDs. Inclusion of these disease-defining variables may restrict the interpretability of the model regarding independent associations with vitamin D. Various thresholds have been used for categorizing vitamin D levels as deficient [21], and the use of different categories may have affected our findings. However, because most of our analyses were based on vitamin D levels rather than categories, the use of different cut-off levels for vitamin D deficiency would not have altered our overall conclusions.

## 5. Conclusions

In conclusion, our study of a large patient cohort did not identify clinically important differences in vitamin D levels or deficiency status between rheumatology patients with inflammatory vs. non-inflammatory diagnoses, nor did we find a statistical association between vitamin D levels and IRD diagnosis. Vitamin D showed no significant association with CRP or RF in linear regression models. Based on these data, we conclude that vitamin D levels should not be interpreted as a proxy for or driver of inflammatory activity in the clinical setting, although its broader biological role in musculoskeletal health remains undisputed.

## Figures and Tables

**Table 1:** Patient characteristics and mean vitamin D levels by diagnosis (N = 4979).

Diagnosis	n	Age, Mean (SD) Years	n (%) Male	Vitamin D, Mean (SD) ng/mL
Inflammatory	1385	58.6 (15.7)	722 (52.1)	26.6 (13.3)
Rheumatoid arthritis	539	61.8 (14.2)	236 (43.8)	26.2 (13.3)
Peripheral SpA	120	47.9 (13.9)	60 (50.0)	28.5 (15.9)
Axial SpA	98	40.7 (14.0)	55 (56.1)	25.5 (13.3)
Connective tissue disease	88	53.4 (15.5)	19 (21.6)	29.1 (13.9)
Vasculitis	51	61.8 (17.0)	22 (43.1)	28.0 (16.2)
Other inflammatory arthritis	196	56.3 (14.3)	172 (87.8)	24.0 (10.5)
Other IRD (e.g., polymyalgia rheumatica)	293	65.6 (12.4)	158 (53.9)	27.4 (12.7)
Non-inflammatory	3594	51.7 (14.6)	1027 (28.6)	27.7 (14.3)

IRD, inflammatory rheumatic disease; SD, standard deviation; SpA, spondyloarthropathy.

**Table 2:** Associations between clinical variables and IRD diagnosis in logistic regression analysis.

Variable	Regression Weight (B)	SE	*p*-Value	Odds Ratio (exp B)
Vitamin D (ng/mL)	0.005	0.003	0.100	1.005
Pain-VAS	−0.005	0.002	0.024	0.995
PtGA-VAS	0.012	0.002	<0.001	1.012
PHQ-2	0.071	0.028	0.010	1.074
SSS	−0.105	0.016	<0.001	0.901
RF titer (U/mL)	0.009	0.002	<0.001	1.009
ACPA titer (U/mL)	0.010	0.001	<0.001	1.010
ANA/ENA+	2.160	0.204	<0.001	8.672
CRP (mg/dL)	0.401	0.059	<0.001	1.493
ESR (mm/h)	0.033	0.004	<0.001	1.034
Leukocytes (×10^3^ per µL)	0.200	0.17	<0.001	1.221
Age (years)	0.012	0.003	<0.001	1.012
Male sex	1.200	0.84	<0.001	3.320
Constant	−4.605	0.229	<0.001	0.010

ACPA, anti-citrullinated peptide antibody; ANA/ENA, antinuclear antibody/extractable nuclear antigen; CRP, C-reactive protein; ESR, erythrocyte sedimentation rate; IRD, inflammatory rheumatic disease; PHQ, Patient Health Questionnaire; PtGA, patient global assessment of disease activity; RF, rheumatoid factor; SE, standard error; SSS, Symptom Severity Scale score for fibromyalgia; U, unit; VAS, visual analog scale (0–100).

**Table 3:** Linear regression model for the impact of selected clinical variables on CRP or RF in the full cohort and in the subgroup of patients with RA. Blue shading indicates statistically significant results.

Dependent Variable	Model Variables	Full Cohort (N = 4975)		RA Patients (N = 539)	
		β Coefficient ^a^ (SE)	*p*-Value	β Coefficient ^a^ (SE)	*p*-Value
CRP	Vitamin D (ng/mL)	<−0.001 (0.001)	0.670	−0.005 (0.006)	0.432
	Pain-VAS	0.002 (0.001)	0.003	−0.001 (0.005)	0.784
	PtGA-VAS	0.002 (0.001)	0.001	0.011 (0.004)	0.006
	PHQ-2	0.018 (0.010)	0.080	−0.036 (0.056)	0.528
	SSS	−0.010 (0.006)	0.110	0.037 (0.034)	0.274
	RF titer (U/mL)	<0.001 (<0.001)	0.974	−0.001 (0.001)	0.348
	ACPA titer (U/mL)	<0.001 (<0.001)	0.477	−0.001 (<0.001)	0.116
	ANA/ENA+	−0.219 (0.088)	0.013	−0.187 (0.402)	0.642
	ESR (mm/h)	0.059 (0.001)	<0.001	0.084 (0.004)	<0.001
	Leukocytes (×10^3^ per µL)	0.073 (0.006)	<0.001	0.096 (0.030)	0.001
	Age (years)	−0.001 (0.001)	0.173	0.006 (0.006)	0.276
	Male sex	0.342 (0.032)	<0.001	0.621 (0.167)	<0.001
RF	Vitamin D (ng/mL)	−0.65 (0.049)	0.187	−0.352 (0.442)	0.427
	Pain-VAS	−0.015 (0.035)	0.667	0.060 (0.343)	0.861
	PtGA-VAS	0.029 (0.031)	0.355	−0.064 (0.298)	0.831
	PHQ-2	0.691 (0.476)	0.147	3.906 (4.119)	0.343
	SSS	−0.493 (0.276)	0.074	−1.846 (2.450)	0.452
	ACPA titer (U/mL)	0.241 (0.008)	<0.001	0.217 (0.029)	<0.001
	ANA/ENA+	3.861 (4.046)	0.340	−39.473 (29.321)	0.179
	CRP (mg/dL)	−0.021 (0.650)	0.974	−2.988 (3.179)	0.348
	ESR (mm/h)	0.143 (0.066)	0.030	0.227 (0.424)	0.592
	Leukocytes (×10^3^ per µL)	0.772 (0.298)	0.009	5.635 (2.197)	0.011
	Age (years)	0.116 (0.047)	0.014	0.096 (0.425)	0.822
	Male sex	−0.973 (1.488)	0.513	−16.933 (12.339)	0.171

^a^ Unstandardized coefficient representing the effect size. ACPA, anti-citrullinated peptide antibody; ANA/ENA, antinuclear antibody/extractable nuclear antigen; CRP, C-reactive protein; ESR, erythrocyte sedimentation rate; PHQ, Patient Health Questionnaire; PtGA, patient global assessment of disease activity; RF, rheumatoid factor; SE, standard error; SSS, Symptom Severity Scale score for fibromyalgia; U, unit; VAS, visual analog scale (0–100).

## Data Availability

The original contributions presented in this study are included in the article. Further inquiries can be directed to the corresponding author.

## Supplementary Materials

The following supporting information can be downloaded at https://www.mdpi.com/article/10.3390/nu18020326/s1. Table S1: Vitamin D status by IRD diagnosis (N = 1385). Figure S1: Correlation coefficients (*r*) for variables tested for their association with inflammatory disease in the logistic regression analysis.

## Abbreviations

The following abbreviations are used in this manuscript:

ACPA	Anti-citrullinated peptide antibody
ANA	Anti-nuclear antibody
BMI	Body mass index
CRP	C-reactive protein
EHR	Electronic health record
ELISA	Enzyme-linked immunoassay
ENA	Extractable nuclear antigen
ESR	Erythrocyte sedimentation rate
IRD	Inflammatory rheumatic disease
PHQ	Patient Health Questionnaire
PRO	Patient-reported outcome
PtGA	Patient assessment of global disease activity
RA	Rheumatoid arthritis
RF	Rheumatoid factor
SD	Standard deviation
SpA	Spondyloarthropathy
SSS	Symptom Severity Scale for fibromyalgia
VAS	Visual analog scale
