# Supplementary material for: Vitamin D Status in Rheumatology Patients with Inflammatory Compared with Non-Inflammatory Diagnoses: Inflammatory and Autoimmune Markers Are Not Associated with Vitamin D Levels
Source: Nutrients. 2026 Jan 20;18(2):326. doi: 10.3390/nu18020326 (PMC12844765; doi:10.3390/nu18020326)
Supplement: Supplementary file 1 [file nutrients-18-00326-s001.zip › nutrients-4079243-supplementary.pdf]

Supplementary Materials

Vitamin D status in rheumatology patients with inflammatory compared with non-inflammatory diagnoses: inflammatory and autoimmune markers are not associated with vitamin D levels

Arne Schäfer, Magdolna Szilvia Kovacs, Axel Nigg, Martin Feuchtenberger

Supplementary Table S1 Vitamin D status by IRD diagnosis (N=1385).

**Diagnosis (n)**	**Vitamin D category, n (%)**			
	**Deficient (<20 ng/mL)**	**Sufficient (20-30 ng/mL)**	**Optimal (>30-70 ng/mL)**	**Elevated (>70 ng/mL)**
Rheumatoid arthritis (n=539)	183 (34.0)	187 (34.7)	161 (29.9)	8 (1.5)
Peripheral SpA (n=120)	38 (31.7)	43 (35.8)	35 (29.2)	4 (3.3)
Axial SpA (n=98)	35 (35.7)	39 (39.8)	23 (23.5)	1 (1.0)
Connective tissue disease (n=88)	22 (25.0)	32 (36.4)	33 (37.5)	1 (1.1)
Vasculitis (n=51)	16 (31.4)	17 (33.3)	16 (31.4)	2 (3.9)
Other inflammatory arthritis (n=196)	78 (39.8)	73 (37.2)	45 (23.0)	0
Other IRD (n=293)	81 (27.6)	109 (37.2)	100 (34.1)	3 (1.0)

IRD, inflammatory rheumatic disease; SpA, spondyloarthropathy

Supplementary Figure 1 Correlation coefficients (r) for variables tested for their association with inflammatory disease in the logistic regression analysis. Moderate and higher correlations (>0.3) are shown in purple. Negative correlations between variables that show positive bivariate correlations, such as CRP and ESR, indicate a suppressor effect due to a strong overlap between variables.

VARI-ABLE	Vit D	Pain-VAS	PtGA-VAS	PHQ-2	SSS	RF	ACPA	ANA/ENA	CRP	ESR	Leuk	Age	Male sex
Vit D	1.00	0.002	0.020	-0.004	-0.016	0.035	-0.012	0.013	0.009	0.054	0.056	-0.048	0.113
Pain-VAS		1.00	-0.583	-0.226	-0.070	0.005	-0.002	0.032	-0.078	-0.034	-0.042	0.022	-0.011
PtGA-VAS			1.00	-0.093	-0.057	0.013	0.014	0.019	-0.022	0.019	0.045	-0.059	0.083
PHQ-2				1.00	-0.477	-0.13	-0.10	0.033	-0.017	0.007	-0.019	-0.037	-0.067
SSS					1.00	0.015	0.016	-0.054	0.029	0.006	-0.041	0.110	0.123
RF						1.00	-0.155	-0.047	0.005	-0.020	0.054	-0.059	0.060
ACPA							1.00	0.026	0.007	0.008	0.035	0.045	0.057
ANA/ENA								1.00	0.043	-0.038	0.116	0.022	0.108
CRP									1.00	-0.493	-0.147	0.018	-0.102
ESR										1.00	-0.014	-0.172	-0.234
Leuk											1.00	-0.079	0.039
Age												1.00	-0.034
Male sex													1.00

ACPA, anti-citrullinated peptide antibody; ANA, antinuclear antibody; CRP, C-reactive protein; ENA, extractable nuclear antigen; ESR, erythrocyte sedimentation rate; Leuk, leukocyte count; PHQ, Patient Health Questionnaire; PtGA. patient global assessment of disease activity; RF, rheumatoid factor; SSS, Symptom Severity Scale score for fibromyalgia; VAS. visual analog scale (0-100); Vit, vitamin.
